# Mechanisms of estrogen deficiency-induced osteoporosis based on transcriptome and DNA methylation

**DOI:** 10.3389/fcell.2022.1011725

**Published:** 2022-10-17

**Authors:** Ziying Xu, Zihui Yu, Ming Chen, Mingming Zhang, Ruijing Chen, Haikuan Yu, Yuan Lin, Duanyang Wang, Shang Li, Ling Huang, Yi Li, Jing Yuan, Pengbin Yin

**Affiliations:** ^1^ Department of Bacteriology, Capital Institute of Pediatrics, Beijing, China; ^2^ Department of Orthopedics, General Hospital of Chinese PLA, Beijing, China; ^3^ National Clinical Research Center for Orthopedics, Sports Medicine and Rehabilitation, Beijing, China; ^4^ The Department of Orthopedic Surgery, Second Affiliated Hospital of Harbin Medical University, Harbin, China; ^5^ School of Traditional Chinese Medicine, Beijing University of Chinese Medicine, Beijing, China

**Keywords:** osteoporosis, estrogen, transcriptome, DNA methylation, 5 mC

## Abstract

Osteoporosis is a disease that impacts the elderly. Low estrogen is related to changes in DNA methylation and consequent alterations in gene expression, leading to a new direction in research related to the pathophysiology of osteoporosis. We constructed an Ovariectomized (OVX) mouse model in our study, and the mouse models had osteoporosis based on the phenotype and methylation levels in the mouse’s bone. Furthermore, the methylation level of the OVX mice was significantly changed compared to that of SHAM mice. Therefore, we performed genome-level analysis on the mouse model using transcriptome and Whole Genome Bisulfite Sequencing (WGBS) by combining the data of two omics and discovered that the changes in gene expression level caused by osteoporosis primarily focused on the decrease of bone and muscle development and the activation of the immune system. According to intersection analysis of methylation and transcriptome data, the differentially expressed genes and pathways are consistent with the differentially expressed methylation locations and regions. Further, the differentially expressed methylation sites were mainly concentrated in promoters, exons, and other critical functional regions of essential differentially expressed genes. This is also the primary cause of gene differential expression variations, indicating that estrogen deficiency might regulate gene expression by altering methylation modification, leading to osteoporosis. We demonstrated the clinical value of methylation modification research, and these findings would improve the current understanding of underlying molecular mechanisms of osteoporosis incidence and development and provide new ideas for early detection and treatment of osteoporosis.

## Introduction

Osteoporosis is a common public health problem that significantly impacts people worldwide. The incidence rate of osteoporosis gradually increases in women over 55 and men over 65 years (Compston, McClung, and Leslie 2019), and OP is twice as frequent in postmenopausal women than in males (Nayak, Nayak, and Mythili 2020). Due to the obvious disease’s insidious nature, most patients are not identified until after a fracture due to the insidious nature of the disease, delaying the optimal time for medical treatment and leading to severe consequences (Brown 2017). Hip fractures, for example, have a 1-year mortality rate of up to 36% (Cosman et al., 2015); and even if they survive, more than half of them are left disabled (Bliuc et al., 2009), causing a lot of stress and suffering to patients and their families. However, the pathophysiology of osteoporosis is still unclear (Wang and McCauley 2016), which makes the early detection, effective prevention, and treatment more challenging. Therefore, osteoporosis has become a tremendous financial burden to the medical and health systems and society, specifically the health of the elderly.

Previous research demonstrated that the dynamic balance of bone mass is maintained by a complicated process of old bone resorption and new bone formation and is regulated by various cell types. According to evidence, estrogen deficiency is a primary factor contributing to osteoporosis regardless of gender (Khosla and Pacifici 2021), and estrogen can promote early osteoblast differentiation and inhibit osteoclast activity (Farr et al., 2019). The decreased estrogen levels following menopause increase osteoclast activity, decrease bone density, increase bone conversion rate, influence calcium deposition, and promote bone ablation, ultimately leading to osteoporosis (Gauthier et al., 2011). Estrogen acts on various cell types and plays a role while performing its regulatory function, but previous studies have shown that estrogen has a higher impact on certain cell groups, including immune (Mohammad et al., 2018) and hepatic cells (Batmunkh et al., 2017). The regulation of estrogen is a significant element in this. Estrogen plays a significant role in intercellular interactions in the bone to maintain its physiological functions, including osteoblast-osteoclast coupling, osteoblast-vascular endothelial cell coupling, osteoclast-vascular endothelial cell coupling, neurovascular coupling, immune cell-osteoclast coupling, and many others. Therefore, estrogen is essential for the normal physiology of bone cells, and the absence of it will cause osteoporosis.

Recently, DNA and RNA methylation modification has become a hot topic in research. 5mC and 5hmC are the most common DNA methylation modifications and comprise most of these alterations. 5mC is a well-studied modification, and its concentration significantly impacts gene expression in tumors and numerous diseases (Madhavan and Nagarajan 2020). The ten-eleven translocation (TET) family of proteases alters 5mC to 5hmC (Tahiliani et al., 2009). Many studies discovered that this modification is significant in embryo development (Cao et al., 2014) and tumor differentiation (Peng et al., 2018). Low concentrations of 5hmC have a poor prognosis and survival rate in malignancies (Chen et al., 2016). Therefore, 5hmC could be utilized as a tumor or disease diagnostic indicator. Previously, we identified that the concentrations of 5mC and 5hmC on DNA influence the occurrence and prognosis of diseases, including cancers (Peng et al., 2018). Simultaneously, methylation changes at multiple gene elements cause an increase or decrease in gene expression, deteriorating the degree of malignancy (Chen et al., 2016).

We constructed an Ovariectomized (OVX) model to mimic postmenopausal osteoporosis in human, and the model was monitored and evaluated using micro-CT, bone index evaluation, immunofluorescence staining, and pathology. Transcriptome and whole-genome bisulfite sequence (WGBS) were used to investigate the molecular mechanism of epigenetic changes in OVX-induced osteoporosis in mice after establishing the osteoporosis phenotype.

## Materials and methods

### Animals and experimental design

8-week-old female C57BL/6J were purchased from the Beijing Vital River Laboratory Animal Technology Co. Ltd. (Beijing, China). The animals were housed in an SFP environment. The mice were ovariectomized or sham-operated after adaptive feeding. All the experimental procedures were approved by the Committees of Animal Ethics and Experimental Safety of the Capital Institute of Pediatrics.

### Micro-CT analysis

Mouse femurs were harvested and all the connected tissues were removed, and the remaining tissue were stored in cold PBS (Gibco). The femurs were scanned with a Inveon MM system (Siemens, Munich, Germany). Briefly, the specimens were scanned at a voltage of 80 kV, a current of 450 μA, and an exposure time of 40 min in each of the 360 rotation steps. Three-dimensional reconstruction was performed by two-dimensional images. Bone mineral density (BMD), bone tissue ratio (BV/TV), bone surface area to volume ratio (BS/BV), bone trabecular number (Tb. N), bone trabecular thickness (Tb. Th), bone trabecular separation (Tb. Sp), and bone trabecular pattern factor (Tb. Pf) were calculated by Inveon Research Workplace (Siemens).

### Hematoxylin and eosin (H and E) staining

Mouse femurs were dissected and incubated in 15% EDTA (EDTA, FREE ACID, Cat#E8040, Solarbio) for decalcification. Then specimens were embedded in paraffin and sectioned into 5 μm slices. After deparaffined and rehydrated, the specimens were stained with hematoxylin and eosin.

### Immunofluorescence

The tibias were dissected and fixed in 4% paraformaldehyde (Macklin) at 4°C for 12 h, and incubated in 15% EDTA (EDTA, FREE ACID, Cat#E8040, Solarbio) for decalcification. The specimens were embedded in OCT and sectioned at 10 μm, then permeabilized with 0.3% Triton for 5 min. After that, the specimens were blocked in 1:10 goat serum for 1 h and then stained at 4°C overnight with primary antibody OSX (sc-393325, Santa Cruz Biotechnology, Mouse monoclonal, 1:100), or anti-5hmC (Active Motif, 39,769, 1:300). Goat anti-mouse IgG H&L Alexa 488 (ab150113, Abcam, 1:100) was used as the second antibody. DAPI (Sigma) was used for nuclear staining.

### Dot blot

Genomic DNA was extracted from tibias using FastPure Cell/Tissue DNA Isolation Mini Kit (Vazyme, DC102-01, China) according to the manufacturer’s instructions. Qubit Fluorometer 4.0 (Life Technology, United States) was used to quantify the DNA concentration. DNA samples were diluted with 10x NaOH and 10 mM Tris at pH 8.5 and then loaded onto a Hybond N+ nylon membrane (GE Health, RPN303B, United States) using a 96-well dot blot apparatus (Bio-Rad, United States). After hybridizing at 80°C for 2 h and being blocked with 5% nonfat milk for 1 h at room temperature, the membrane was incubated in anti-5mC antibodies (ZYMO RESEARCH, #A3001-200, 1:5000) and anti-5hmC (Active Motif, 39,769, 1:10,000) at 4 °C overnight and visualized by chemiluminescence. To ensure equal loading, the membrane was then stained with methylene blue.

### Tissue RNA isolation and rRNA removal RNA-seq

Total RNA of tibias extraction using FastPure Cell/Tissue Total RNA Isolation Kit V2 (Vazyme, RC112-01, China) according to the manufacturer’s instructions. A Qubit Fluorometer (Invitrogen, United States) was used for RNA integrity determination and quantification. The rRNA Removal Kit (Vazyme, N406-01, China) and VAHTS universal V8 RNA-seq Library Preparation Kit for Illumia (Vazyme, NR605-01, China) were used to construct stranded RNA-seq libraries according to the manufacturer’s instructions.

### Tissue DNA isolation and WGBS

Genomic DNA of tibias extraction using FastPure Cell/Tissue DNA Isolation Mini Kit (Vazyme, DC102-01, China) according to the manufacturer’s instructions. After quantification through Qubit, 1 μg gDNA underwent bisulfite conversion using the EpiArt DNA Methylation Bisulfite Kit (Vazyme, EM101-01, China). 100 ng bisulfite-treated DNA was quantification through Qubit, then end-repaired, A-tailed, and ligated to methylated adaptors following the manufacturer’s instructions.

### Statistical analyses

All data were expressed as the mean ± *s*.d. Statistical differences among groups were analyzed by one-way analysis to determine group differences. All statistical analyses were performed with GraphPad software, version 9.3.1.471. Statistical differences between the two groups were determined by the Student’s t-test. *p* < 0.05 was considered statistically significant.

### WGBS data analysis

The raw reads were trimmed through Trimmomatic (Trimmomatic-0.32) software using the default parameters. The clean reads were aligned to the mm10 genome using Bismark v0.14.3 and Bowtie2 v2.0.0. The Samtools v0.1.19 was used to process BAM files for sorting, merging, duplicate removal, and indexing. The average bisulfite conversion rate of unmethylated Lambda DNA was 99.9% on average. CpGs, differentially methylated loci (DMLs), and differentially methylated regions (DMRs) were called using DSS v_2.30.1 with default parameters (Park and Wu 2016). To detect differential methylation, statistical tests were conducted at each CpG site, and the differential methylation loci (DML, *p* = 0.001) were called. Differential methylation regions (DMRs) detection was based on the DML results (delta = 0.01, *p* < 0.01). Finally, regions with differences in methylation >0.2 are identified as DMRs in this study. We used the Bioconductor package TxDb. Mmusculus UCSC. mm10.knownGene as reference. The annotation of DMRs was performed in R with the Bioconductor package ChIPseeker (Yu, Wang, and He 2015).

### RNA-seq data analysis

We downloaded the human liver transcriptome data Array Express with the accession code e-MTAB-6814. The reads from mice data were aligned against mm10 genome assembly with hisat 2.1.0. SAM files were sorted and converted to BAM with samtools v1.4. Reads with QS < 20 were excluded. For each sample, unique map reads with map quality score ≥20 were reserved for subsequent analyses. HT Seq Python package (version 0.9.1) was used to count the number of reads of a unique map for each gene. R-package DEseq2 was used to determine the differentially expressed genes; fold-change cutoff 1.0, *p*-value cut-off 5 × 10^−2^.

### Functional enrichment analysis

Gene ontology (GO) was analyzed with ClusterProfiler, an R package that analyzes and visualizes functional profiles (GO or KEGG) of genes and gene clusters. GO terms with *p* < 0.05 were considered to be statistically significant.

### Bisulifite-specific PCR

Primers of differentially methylated regions (DMRs) of *Ppargc1a*, *Usp2*, *Cd5* and *Cd6* were designed by MethPrimer 2.0 algorithm (http://www.urogene.org/methprimer2/) and shown Below. They both included Ts corresponding to non-CpGs to prevent amplification of nonbisulfifite-converted DNA. The PCR reaction system was done using EpiArt HS Taq Master Mis (Vazyme, EM202-01, China) according to the manufacturer’s instructions. The PCR product was visualized on a 1% agarose gel. Table 1 Primers of Bisulifite-specific PCR.

### Real-time PCR

One ug RNA were reverse transcribed using HiScript II Q Select RT SuperMix for qPCR (+ gDNA wiper) (Vazyme, R323, China). Real-time RT-PCR was performed on the CFX96 Touch system detection system (Bio-Rad) using ChamQTMSYBRqPCR Master Mix (Vazyme, Q711, China) and the primers listed below. Gene expression analysis was performed using the comparative 2-^ΔCt^ method with GAPDH for normalization. Table 2 Primers of Real-Time PCR.

## Results

### OVX mice displayed an osteoporotic phenotype, as revealed by micro-CT

**FIGURE 1:**
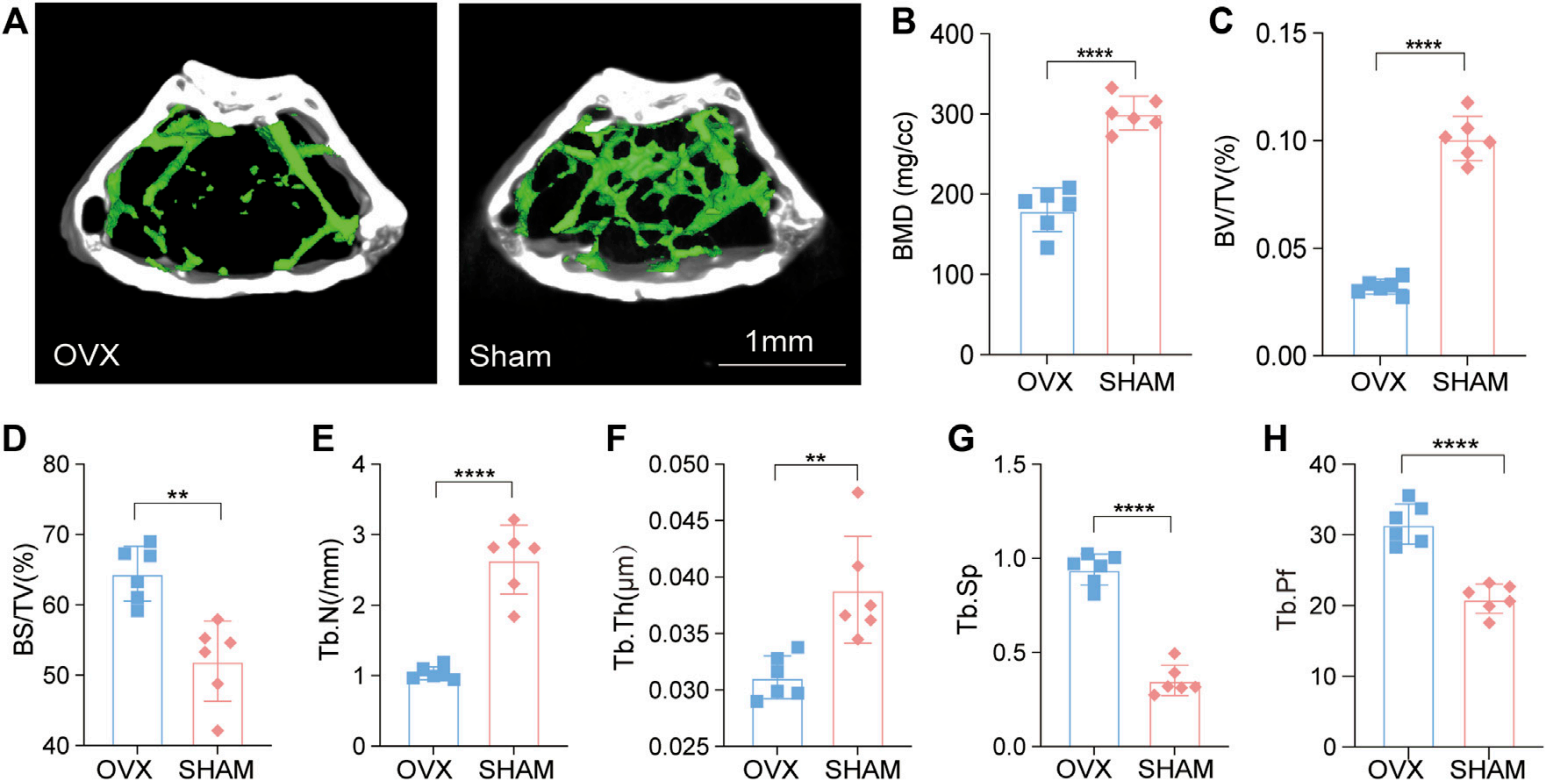
Micro-CT revealed an osteoporotic phenotype in OVX mice. **(A)** OVX and Sham mice micro-CT imaging; **(B–H)**. Bone metrology comparison: BMD, BV/TV, BS/BV, Tb. N, Tb. Th, Tb. Sp, Tb. Pf (*n* = 6, *p, 0.05, **p, 0.01, ***p, 0.005, ****p, 0.001, *t*-test).

The degree of osteoporosis in C57 mice was evaluated in many ways after stabilizing the mice to define their phenotypes. Micro-CT scans of the femur bone of OVX and SHAM mice were performed, and an in-depth analysis of the images revealed that the femur cavity of OVX mice was significantly prominent, and the trabecular bone was sparse and disorganized (Figure 1A). According to quantitative analysis, bone mineral density (BMD), bone tissue ratio (BV/TV), bone surface area to volume ratio (BS/BV), bone trabecular number (Tb. N), and bone trabecular thickness (Tb. Th) in the OVX group were lower than the SHAM group. Further, bone trabecular separation (Tb. Sp) and bone trabecular pattern factor (Tb. Pf) were also increased. The metabolism of the OVX mice’s bones was imbalanced, resulting in decreased bone mass and impaired microarchitectures. These results indicated that OVX mice developed osteoporosis (Figures 1B–H).

### The pathological results of OVX mice demonstrated severe osteoporosis and modifications in DNA methylation levels

**FIGURE 2:**
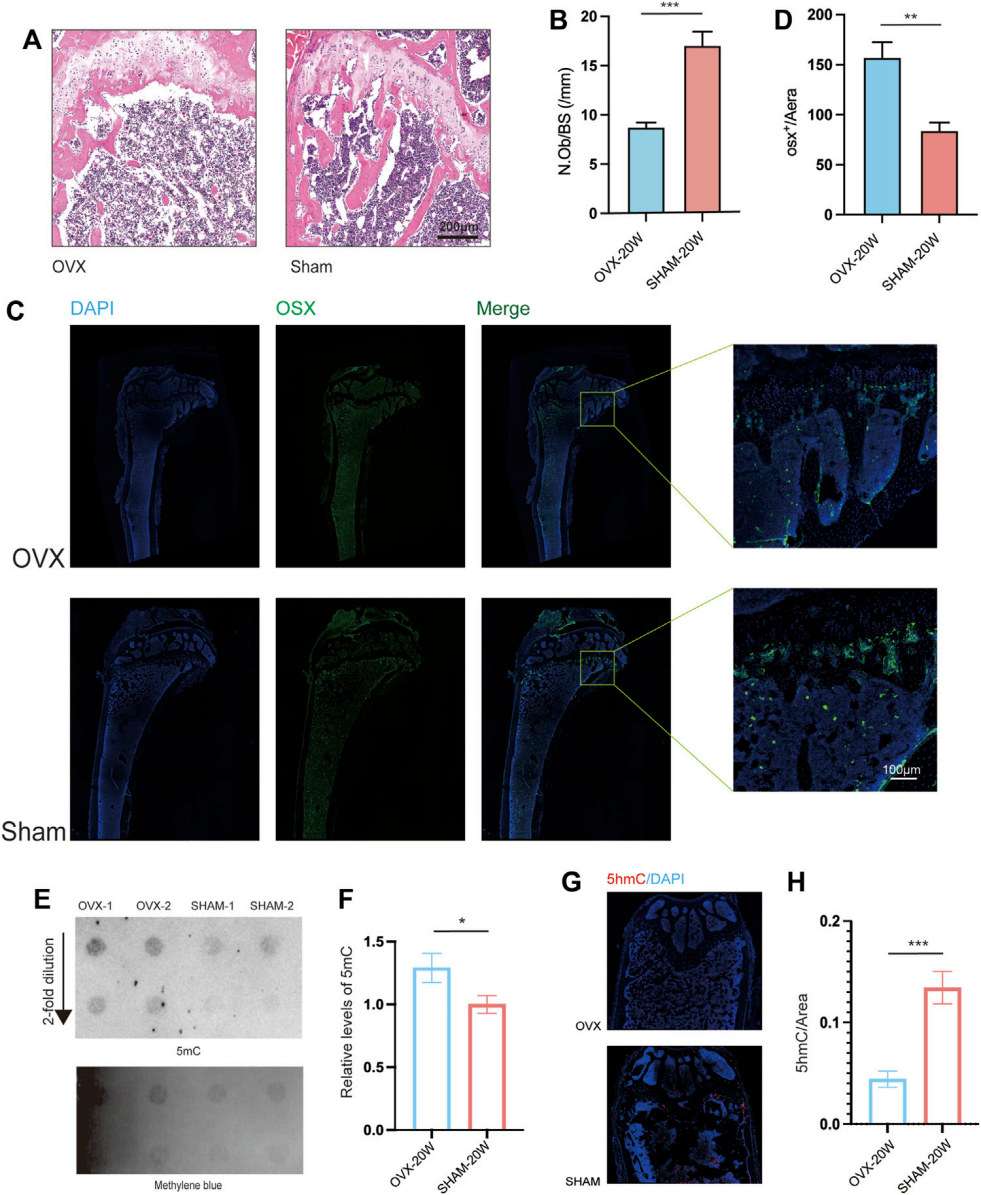
Histopathological and Dot Blot analyses revealed osteoporosis and increased DNA methylation levels in OVX animals. **(A)** Bone HE sections from OVX and SHAM mice. Scale bar: 200 μm. **(B)** N.Ob/BS quantification from . **(A)** (*n* = 6, ***p, 0.005, *t*-test). **(C)** Representative immunofluorescence images of OSX. **(D)** Quantitation of OSX+/Area from a distance of 100 μm (C) (*n* = 6, **p, 0.01, *t*-test). **(E)**. Dot blot assays of 5 mC levels in OVX and SHAM mice’s bone;**(F)**. Dot-blot semiquantitative (*n* = 6, **p, 0.01, *t*-test).**(G)**. Representative immunofluorescence images of 5hmC;**(H)**. Quantitation of 5hmC from (*n* = 6, ***p, 0.005, *t*-test).

Furthermore, we stained the bone samples of mice in the OVX and SHAM groups using Hematoxylin and eosin (H&E) stain (Figure 2A). The number of osteoblasts/bone surface (N.Ob/BS) in the OVX group was significantly lower than in the SHAM group (Figure 2B). We also stained mouse femur tissue slices with immunofluorescence (Figure 2C), and the results revealed that the OVX group mice had less Osterix-positive cells in the epiphyseal region than the SHAM group mice (Figure 2C). The osteogenic ability of mice in the OVX group was significantly lower than that of mice in the SHAM group (Figure 2D).

Many studies found that aberrant DNA methylation levels in tissues associated with disease agenetic alterations. Consequently, dot blot assays on bone DNA from OVX and SHAM mice were conducted. The results showed that the gray value of 5 mC spots in the DNA of OVX mice was higher than that of SHAM mice (Figure 2E), and quantitative analysis confirmed a significant difference in the 5 mC level. (Figure 2F). Furthermore, mouse bone slices were stained with 5 hmC immunofluorescence (Figure 2G), and the level of 5 hmC in SHAM mice was significantly higher than in OVX mice based on quantitative results.

### Bone-function genes in OVX and SHAM mice were associated with bone development and immunity - Transcriptome analysis

**FIGURE 3:**
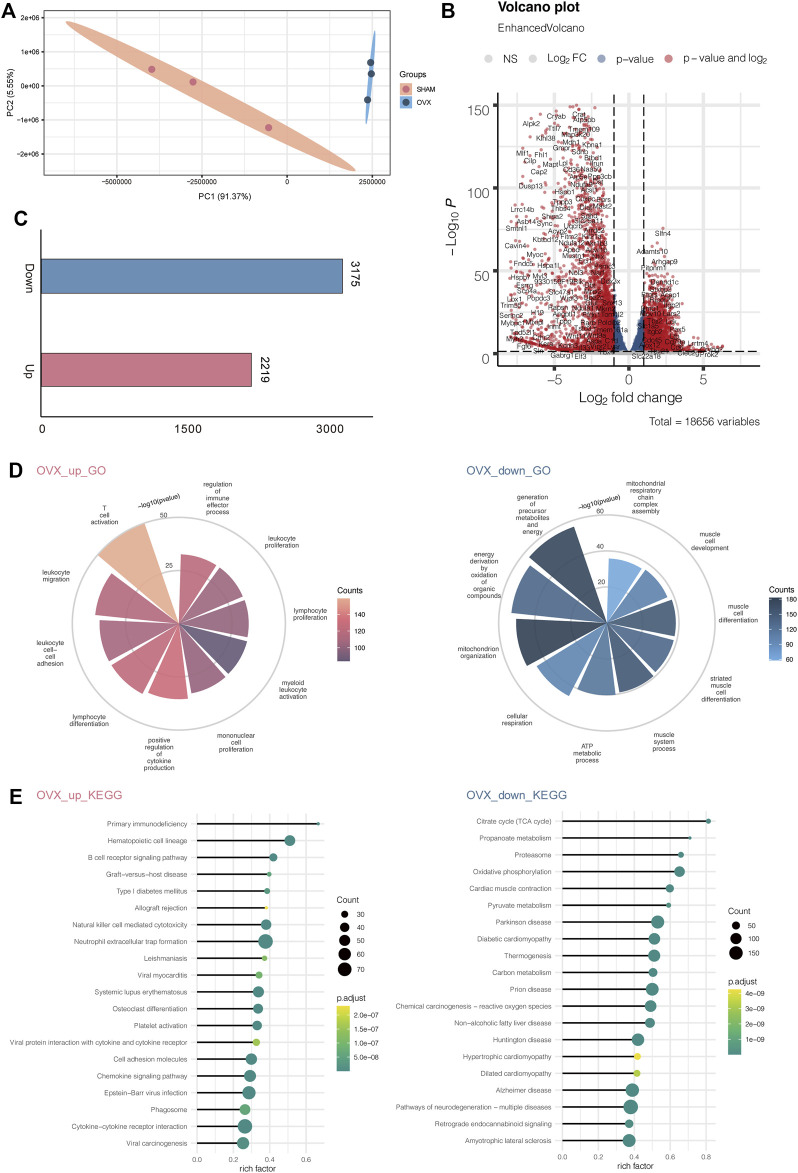
OVX mice’s transcription profile differed significantly from SHAM mice'. **(A)** PCA gene expression profile in OVX and SHAM mice. **(B)** Volcanic map showing the differentially expressed genes of OVX and SHAM mice. **(C)** Numbers of up-and down-regulated genes. **(D)** The enriched GO terms of RNA-seq data OVX and SHAM mice. **(E)** The enriched KEGG pathways of RNA-seq data OVX and SHAM mice.

Total RNAs of bone tissue from 20 weeks old OVX and SHAM mice were prepared, and transcriptomic sequencing analysis was performed to remove ribosomal RNAs. We used three mice in each group, and the samples within and between groups had high correlations, indicating that the modeling was effective (Supplementary Figure S1). We selected different expression genes in the dataset of OVX and SHAM mice for Principal Component Analysis (PCA) (Figure 3A; Tables 1, 2) and found that the genes of bone tissues in the two groups were significantly different. The volcanic map depicts the differentially expressed genes between the two groups (Figure 3B).

**TABLE 1:** Primers of Bisulifite-specific PCR.

Genes		Sequences (5′ to 3′)
*Ppargc1a*	Sense	TGA​AAT​ATT​GTT​TAG​GAG​GTA​GAG​G
	Antisense	AGA​ACC​AGG​ATA​AGA​GGT​AAG​CG
*Usp2*	Sense	ATT​GGG​ATA​TAT​AGA​TAG​AGA​AGA​TAA​GAT
	Antisense	AAA​AAA​TAC​TTT​CTT​AAC​ACC​ACT​AAA​AA
*Cd5*	Sense	TAA​TTA​GGA​AAT​AGA​GAG​AAA​AAT​TAA​GAT
	Antisense	AAT​CTC​AAA​ACT​TTA​CTA​CAC​AAC​CAA​A
*Cd6*	Sense	TTT​TTG​AGA​TGT​TAA​AGG​GAA​AA
	Antisense	TAA​ATT​CTA​AAA​CAT​TCT​CAC​AT

**TABLE 2:** Primers of Real-Time PCR.

Genes		Sequences (5′ to 3′)
*GAPDH*	Sense	AGG​TCG​GTG​TGA​ACG​GAT​TTG
	Antisense	GGG​GTC​GTT​GAT​GGC​AAC​A
*Ppargc1a*	Sense	TAT​GGA​GTG​ACA​TAG​AGT​GTG​CT
	Antisense	CCA​CTT​CAA​TCC​ACC​CAG​AAA​G
*Usp2*	Sense	TGG​GGC​TTC​TGC​TCA​ACA​AAG
	Antisense	TCG​TGG​GGG​ACC​CTA​TAA​AAC
*Cd5*	Sense	CCT​TGC​CAA​TTC​GAT​GGG​AG
	Antisense	CTG​GGC​CGT​TGT​TTT​CTG​GA
*Cd6*	Sense	GGA​GGG​CTA​CTG​CAA​TGA​TCC
	Antisense	GTG​AGG​GGA​CTC​TTC​TCA​GAA​T

Then, we calculated the number of up-regulated genes and found 2219 up-regulated genes and 3175 down-regulated genes (Figure 3C). Next, we enriched the GO pathway of these differentially expressed genes and observed that the up-regulated genes were mostly enriched in T cell activation, leukocyte migration, leukocyte cell-cell adhesion, lymphocyte differentiation, and positive regulation of cytokine production. These immune system activation pathways suggested that OVX mice had a prominent response to osteoporosis in line with the clinical findings. Down-regulated genes were enriched in the GO pathway through the generation of precursor metabolites and energy, energy derivation by oxidation of organic compounds, mitochondrion organization, cellular respiration, and ATP metabolic process. These pathways were linked to decreased energy metabolism and bone and musculoskeletal development, indicating that osteoporosis causes aberrant bone and muscle cell development and reduces energy metabolism (Figure 3D).

Simultaneously, we investigated the KEGG pathway, including primary immunodeficiency, hematopoietic cell lineage, B cell receptor signaling pathway, graft−*versus*−host disease, and type I diabetes mellitus. The citrate cycle (TCA cycle), propanoate metabolism, proteasome oxidative phosphorylation, and cardiac muscle contraction were among the KEGG pathways enriched by down-regulated genes (Figure 3F).

### Methylation changes in osteoporosis in OVX mice

**FIGURE 4:**
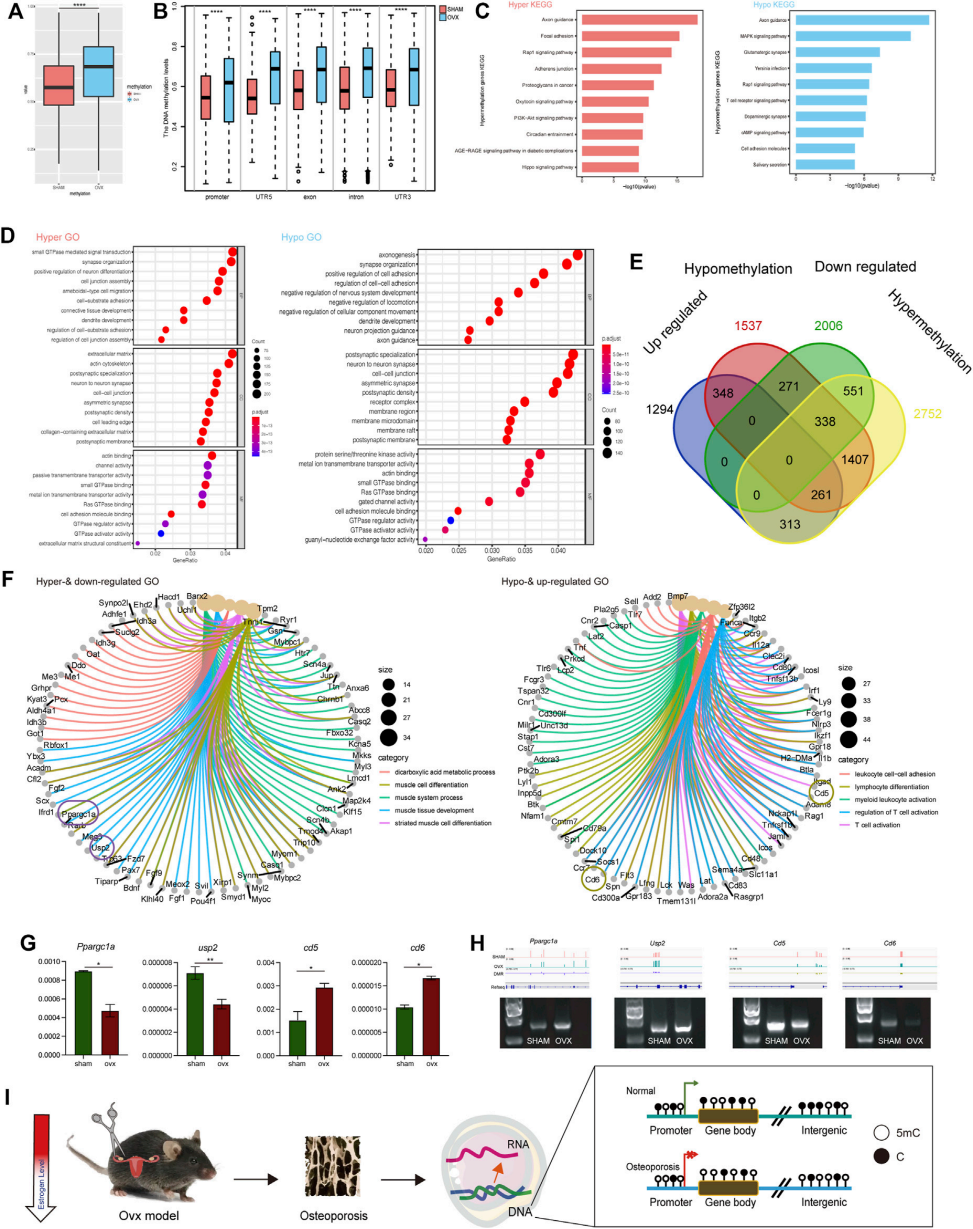
OVX mice’s transcription profile differed significantly from SHAM mice. **(A)** The whole DNA methylation values of OVX and SHAM mice. ****p,0.0001 (*t*-test). **(B)** The DNA methylation levels of DMRs at indicated genomic regions in OVX and SHAM mice. ****p,0.0001 (*t*-test). **(C)** KEGG pathways of hyper- and hypo-methylation genes. **(D)** GO term of hyper- and hypo-methylation genes. **(E)** Venn diagram displays up-regulated and hypo-methylation and down-regulated and hyper-methylation genes in OVX and SHAM mice. **(F)** GO term of up-regulated and hypo-methylation and down-regulated and hyper-methylation genes. **(G)** RT-PCR analysis of the expression of mRNA -related genes (Usp2, Ppargc1a, Cd5, Cd6). Expression level presented as means ± SD (n = 3, **p, 0.01, *t*-test, vs SHAM). **(H)** The DNA methylation pattern in genomic loci of *Usp2, Ppargc1a, Cd5, Cd6.* Agarose gel shows amplicons from methylated primer pairs of the DMRs of these four genes. **(I)** A schematic chart of methylation modification in OVX mice.

In previous phenotypic studies, we found that the methylated 5 mC level in OVX mouse bone DNA was significantly increased, while the methylated 5hmC level was significantly decreased. Therefore, we analyzed DNA methylation changes in the samples at the single-base level using whole-genome methylation sequencing. Results showed that the overall methylation level of OVX mice was significantly higher than that of the SHAM group (Figure 4A), which was consistent with previous dot blot results (Figure 2E). Meanwhile, we checked the methylation level of gene elements in the two groups and found that regardless of the promoter, 5 ′UTR, exon, intron, and 3′ UTR regions, the methylation level of the OVX group was significantly higher than that of the SHAM group (Figure 4B).

In the KEGG pathway, we enriched the genes with hypermethylation (Supplementary Table S3) and hypomethylation (Supplementary Table S4) by measuring and evaluating the differentially methylated genes. Axon guidance, focal adhesion, RAP1 signaling pathway, adherents junction, and proteoglycans in cancer were the primary enriched pathways. It indicated that hypermethylated genes were more tightly connected to disease occurrence. These pathways are associated with tumorigenesis, and it has also been reported that tumorigenesis is significantly associated with osteoporosis (Drake 2013). There were more hypermethylated genes than disease-related axon guidance, MAPK signaling pathway, Glutamatergic synapse, *Yersinia* infection, RAP1 signaling pathway, and other related pathways (Figure 4C).

Simultaneously, we performed GO pathway enrichment for differentially methylated genes, and the enriched GO terms for hypermethylation and hypomethylation were presented in Figure 4D. Previous studies reported hypomethylation generally promotes gene expression while hypermethylation inhibits its expression (Gyorffy et al., 2016). We investigated the intersection genes: upregulated and hypomethylated genes and downregulated and hypermethylated genes. We conducted GO enrichment on the intersecting genes in methylation (Figure 4E) and analyzed the top five GO pathways and associated genes (Figure 4F). The upregulated and hypomethylated pathways were leukocyte cell-cell adhesion, lymphocyte differentiation, myeloid leukocyte activation, regulation of T cell activation, and T cell activation. The downregulated and hypermethylated pathways were the dicarboxylic acid metabolic process, muscle cell differentiation, muscle system process, muscle tissue development, and striated muscle cell differentiation. RT-PCR revealed that *Usp2 and Ppargc1a* were downregulation in OVX mice, whereas *Cd5* and *Cd6* were high expression in OVX mice than that in SHAM mice (Figure 4G). We investigated these differentially expressed genes and found that *Usp2, Ppargc1a, Cd5,* and *Cd6* were the differentially expressed genes. These genes were closely related to osteoporosis and were hypermethylated or hypomethylated in primary gene element regions (Figure 4H). The verification results of bisulfite-specific PCR showed that the methylation modification of related DMR regions was consistent with the track in Figure 4H (Figure 4G). In summary, estrogen deficiency causes osteoporosis leading to significant changes in gene expression in bone cells, induced mainly by modifications in DNA methylation (Figure 4I).

## Discussion and conclusion

Many studies reported that the degree of genome methylation modification is directly associated with the occurrence, development, and prognosis of aging (Unnikrishnan et al., 2019) and diseases (Constancio et al., 2020). Furthermore, the influence of methylation enrichment at gene element regions on gene expression regulation has become a hot research topic (Li, Chen, and Lu 2021). Osteoporosis is an age-related disease, and epigenetic modifications in osteoporosis have received considerable attention recently, but the precise mechanisms are yet to be uncovered. In this study, the methylation modifications in a mouse model of osteoporosis were thoroughly discussed. The levels of total DNA methylation 5mC and 5hmC in both groups were significantly increased. The samples were subjected to transcriptome and WGBS analysis, comprehensively illustrating the connection between methylation level changes and significant gene expression variations.

Many studies reported that the degree of genome methylation modification is directly associated with the occurrence, development, and prognosis of aging (Unnikrishnan et al. 2019) and diseases (Constancio et al. 2020). Furthermore, the influence of methylation enrichment at gene element regions on gene expression regulation has become a hot research topic (Li, Chen, and Lu 2021). Osteoporosis is an age-related disease, and epigenetic modifications in osteoporosis have received considerable attention recently, but the precise mechanisms are yet to be uncovered. In this study, the methylation modifications in a mouse model of osteoporosis were thoroughly discussed. The levels of total DNA methylation 5mC and 5hmC in both groups were significantly increased. The samples were subjected to transcriptome and WGBS analysis, comprehensively illustrating the connection between methylation level changes and significant gene expression variations.

The majority of epigenetic studies in osteoporosis were mainly focused on noncoding RNAs as post-transcriptional regulators, and few articles analyzed the role of DNA methylation in osteoporosis pathogenesis (Fittipaldi et al., 2020). Besides, much less is known about the effects of methylation changes on overall gene expression profile by combining the data of two omics (Visconti et al., 2021) In this study, the methylation map of the whole genome of two groups of mice was obtained using WGBS at the single-base level to demonstrate the influence of DNA methylation on osteoporosis, and the OVX group had a high overall methylation level (Figures 4A,B). Then, we examined hypomethylation and upregulated expression genes and hypermethylation and downregulated expression genes (Figure 4E). The upregulated and hypomethylated pathways were leukocyte cell-cell adhesion, lymphocyte differentiation, myeloid leukocyte activation, regulation of T cell activation, and T cell activation. The relevance of immune cells and estrogen deficiency has been demonstrated in several studies. For instance, Pacifici et al. found that estrogen deficiency expanded intestinal Th17 cells and TNF^+^ T cells, and then migrated from the gut to the bone marrow, leading to enhanced cytokine production and bone loss (Yu et al., 2021; Li et al., 2016). The downregulated and hypermethylated pathways were the dicarboxylic acid metabolic process, muscle cell differentiation, muscle system process, muscle tissue development, and striated muscle cell differentiation. Previous studies demonstrated that estrogen deficiency led to a decrease in energy expenditure and an increase in adipose tissue and insulin resistance, which were deemed as characteristics of metabolic syndrome (Mauvais-Jarvis, Clegg, and Hevener 2013; Cavalcanti-de-Albuquerque et al., 2014). Consistent with the current knowledge, those metabolic disturbances reflected in our study further supported an underlying mechanism related to energy utilization under the estrogen deficiency condition.

Further, we selected genes related to osteoporosis expression with significant methylation modifications. We discovered *Usp2*, a ubiquitin-specific protease essential for TNF-alpha, and used it to activate NF-kB signaling. Previous studies indicated that *Usp2* is involved in the occurrence and progression of tumors and many other diseases (Zhu and Gao 2017). For bone, *Usp2* is reported required for the PTH-induced osteoblast proliferation (Shirakawa et al., 2016). However, the specific relationship between *Usp2* and osteoporosis remains unknown. In our study, we confirmed that *Usp2* expression was down-regulated in osteoporosis mice due to hypermethylation. Besides, *Ppargc1a*, a transcriptional coactivator that regulates genes involved in energy metabolism, is encoded by this gene. Its protein interacts with PPAR gamma, allowing it to interact with many transcription factors. It is essential for the regulation of energy metabolism, bone remodeling, and high energy expenditure of the skeleton (Yu et al., 2018). Studies demonstrated that osteocyte-targeted deletion of PPAR gamma in mice improved glucose homeostasis and inhibited hepatic steatosis development, whereas bone frailty was partially prevented in terms of bone strength (Brun et al., 2017). In this study, we elucidated the gene expression profiles related to energy metabolism under osteoporosis conditions and further revealed the epigenetic mechanism of PPAR in bone remodeling.

Further, we identified *Cd5* and *Cd6*, as members of the SRCR superfamily of scavenger receptors. Members of this family are membrane-anchored proteins primarily expressed in immune system cells and are secreted by lymphocyte populations (T cells, and B cells). The excessive expression of these genes has been related to bone and muscle diseases (Nasr et al., 2011; Hou et al., 2021). Several studies have indicated a close relationship between T cells and osteoporosis since T cells were major stimulators of osteoclastogenesis by increasing the production of so-called bone-resorbing cytokines (Pietschmann et al., 2016). From this study, we found activated T cells and upregulated *Cd6* expression in OVX mice, and this change was regulated by DNA methylation. Besides, another study focusing on *CD5* ^*+*^ B cells revealed that the percentages of *CD5* ^*+*^ B cells were positively correlated with the serum levels of the bone resorption marker *CTX-1* in a cross-sectional cohort of RA patients (Engelmann et al., 2015). Given that the possible functional link and driving mediators still need to be identified, in this study, our data hinted that B cells might play pivotal roles in the microenvironment of bone marrow, and participated in the bone remodeling process. Furthermore, the DMR regions of these genes are primarily found at the promoters, exons, and other critical regions, indicating that methylation levels in these areas significantly impact gene expression.

However, our study still has several flaws. Dot blot analysis revealed that the amount of DNA methylation 5 mC in the OVX group was substantially higher (Figures 2E,F). When osteoporosis is compared to normal tissues, gene alterations are the primary driver of pathological changes in organizational structure, and the DNA methylation landscape reveals an enhanced phenotype in the disease state (Skvortsova, Stirzaker, and Taberlay 2019). We found that the amount of 5hmc in the OVX group was reduced when tissue slices were stained for 5hmc (Figures 2G,H). Some studies reported that a change in the 5hmc pattern could lead to osteoporosis (Grandi and Bhutani 2021), and 5hmC has been extensively reported as a significantly reduced marker in malignancies (Ge et al., 2018).

The single base methylation obtained by WGBS is a mixture of 5hmC and 5 mC (Jeong, Guzman, and Goodell 2017), and the 5hmC level covers 5 mC levels of some genes. So, in addition to sequencing analysis of 5 hmC, other sequencing methods, such as oxBS or ACE-seq, are required. However, due to the difficulty of applying these techniques to bone tissue samples, we used optimization methods to perfect the DNA methylation pattern of osteoporosis to explain better the reason for the difference in gene expression in osteoporosis.

## Data Availability

The datasets presented in this study can be found in online repositories. The names of the repository/repositories and accession number(s) can be found below: China National Center for Bioinformation/Beijing Institute of Genomics, Chinese Academy of Sciences (GSA: CRA007214) https://ngdc.cncb.ac.cn/gsa/browse/CRA007214

## Supplementary material

SUPPLEMENTARY FIGURE S1 Heat map of gene expression correlation between OVX and SHAM. Correlation and clustering of homologous gene expression profiles of mice and rats.
