# Supplementary figures and images for: Mechanisms of estrogen deficiency-induced osteoporosis based on transcriptome and DNA methylation
Source: Front Cell Dev Biol. 2022 Oct 17;10:1011725. doi: 10.3389/fcell.2022.1011725 (PMC9618684; doi:10.3389/fcell.2022.1011725)

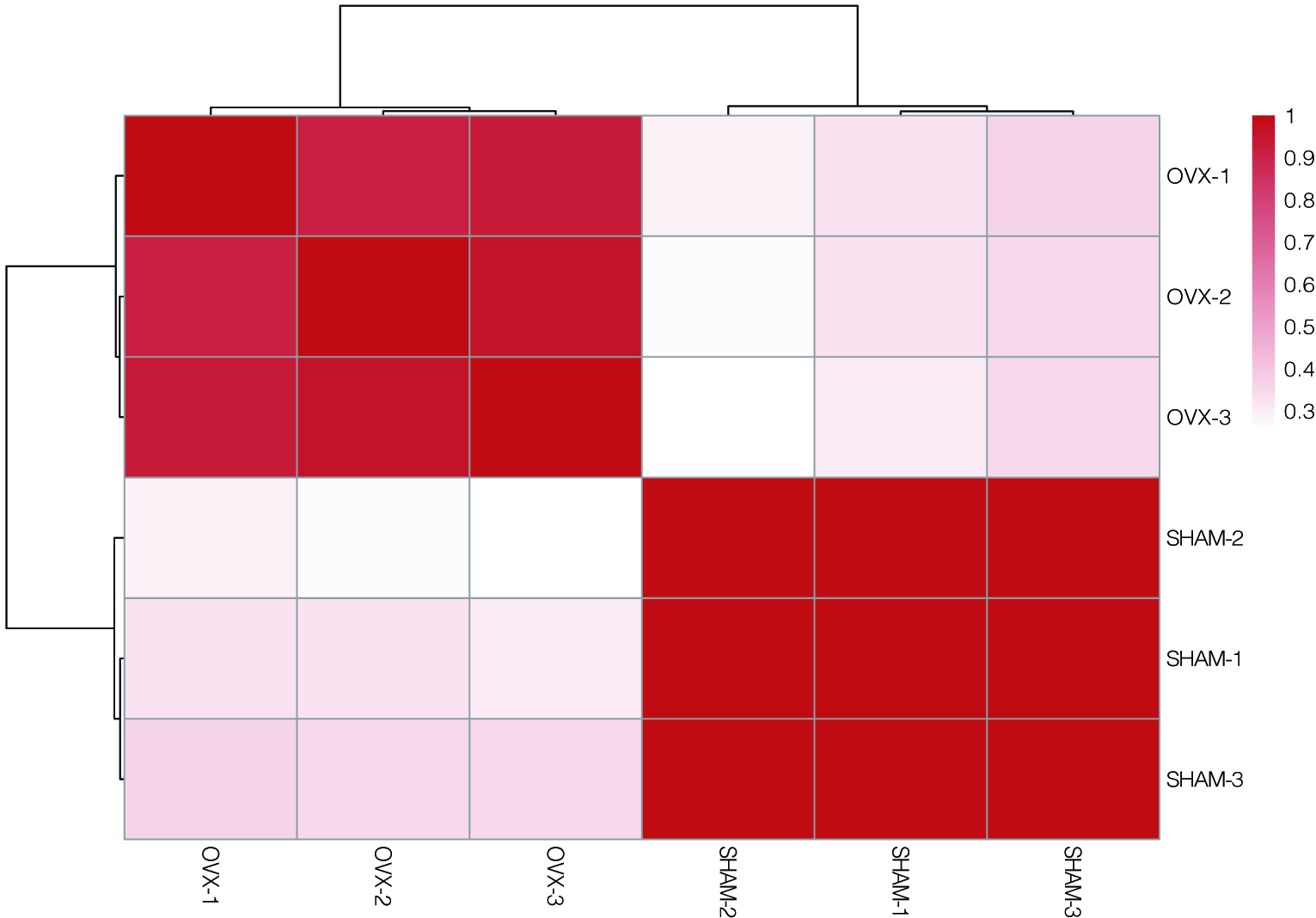

Supplement: Supplementary file 3 [file Image1.TIF]
